# Resistive Switching Characteristics of Li-Doped ZnO Thin Films Based on Magnetron Sputtering

**DOI:** 10.3390/ma12081282

**Published:** 2019-04-18

**Authors:** Xiaofeng Zhao, Yi Li, Chunpeng Ai, Dianzhong Wen

**Affiliations:** Key Laboratory of Electronics Engineering, College of Heilongjiang Province, Heilongjiang University, Harbin 150080, China; 2171256@s.hlju.edu.cn (Y.L.); 2011026@hlju.edu.cn (C.A.); wendianzhong@hlju.edu.cn (D.W.)

**Keywords:** Pt/Ag/ZnO:Li/Pt/Ti memory device, Li-doped ZnO thin films, resistive switching characteristics, magnetron sputtering

## Abstract

A kind of devices Pt/Ag/ZnO:Li/Pt/Ti with high resistive switching behaviors were prepared on a SiO_2_/Si substrate by using magnetron sputtering method and mask technology, composed of a bottom electrode (BE) of Pt/Ti, a resistive switching layer of ZnO:Li thin film and a top electrode (TE) of Pt/Ag. To determine the crystal lattice structure and the Li-doped concentration in the resulted ZnO thin films, X-ray diffraction (XRD) and X-ray photoelectron spectroscopy (XPS) tests were carried out. Resistive switching behaviors of the devices with different thicknesses of Li-doped ZnO thin films were studied at different set and reset voltages based on analog and digital resistive switching characteristics. At room temperature, the fabricated devices represent stable bipolar resistive switching behaviors with a low set voltage, a high switching current ratio and a long retention up to 10^4^ s. In addition, the device can sustain an excellent endurance more than 10^3^ cycles at an applied pulse voltage. The mechanism on how the thicknesses of the Li-doped ZnO thin films affect the resistive switching behaviors was investigated by installing conduction mechanism models. This study provides a new strategy for fabricating the resistive random access memory (ReRAM) device used in practice.

## 1. Introduction

To the best knowledge, the resistive random access memory (ReRAM) has been considered as a promising candidate for next generation nonvolatile memory (NVM) devices [1], due to their relatively excellent properties including small threshold voltage [2], low power consumption, fast switching speed [3], simple structure design and high storage density [4], etc. Currently, the resistive switching behaviors have been reported in a lot of materials, such as complex oxides [5,6], organics [7] and binary metal oxides [8,9,10], etc. However, the complex fabrication technology of multicomponent oxides limits its development due to the difficult controlling doping and uneasy integration of ReRAM. Furthermore, it is hard to design the molecular structure and improve the stability of the ReRAM using organic materials as function layer, such as long retention and excellent endurance. Comparing with that, the binary metal oxides have been common materials for fabricating ReRAM attributing to their simple preparing process, compatibility with integrated circuit processes, and easy to dope [11], etc. Recently, the fabrication of the metal oxide memristors mainly concentrates in ZrO_2_ [12], TiO_2_ [13], NiO [14], HfO_2_ [15] and ZnO [16], etc.

Among these binary metal oxides, ZnO used as a common material in semiconductor ReRAM has attracted more attention attributing to its excellent electrical properties, simple preparation, complementary metal-oxide-semiconductor transistor (CMOS) technology compatibility [17]. For example, Chang et al. proposed a Pt/ZnO/Pt device to realize a reversible and steady bipolar resistive switching behavior with a narrow dispersion of resistance states and a switching voltage [18]. Meanwhile, Wang et al. investigated the effects of different top metal electrodes (including Ag, Ti and Pt) on the resistive switching performance of metal/ZnO/Pt RRAM cells [19]. Nevertheless, most of the pure ZnO-based ReRAMs have low ON/OFF current ratio, poor resistive switching voltage repeatability and low endurance, which limits their applications of the ZnO-based resistive switching devices in the ReRAM from meeting the demand of high performance memristors. Due to the existence of the crystal lattice defects in the ZnO thin film, it is inevitable to form an n-type semiconductor. Utilizing impurity doping makes it possible for compensating the above introduced vacancies, and the properties of ZnO films can be improved significantly [20]. Increasing demand for the ZnO-based material with small amount of lattice defects have triggered a particular research attention to ZnO with doping impurities [20,21] having important effects on the physical properties of the II-VI oxide semiconductors such as electrical resistivity, piezoelectricity and crystal structure, etc. [22]. Especially, Li-doped ZnO thin film-based resistive switching devices have been a research hotspot due to their excellent performances superior to the other doping materials. For example, in 2016, Y. Kafadaryan et al. proposed an Ag/ZnO:Li/SnO_2_:F resistive switching device, which achieves a positive voltage pulse of 3.5 V, quickly switching time of 20 ms and a high repeatability up to 120 s [23]. In 2018, A. S. Igityan et al. proposed a structure of Au/Li_10_ZnO/Li_1_ZnO/LaB_6_ of resistive switching device with a low resistance ratio of 10, high data storage time more than 3 h and more switching cycles up to 350 cycles [24]. On the basis of that, without a robust strategy to control stability and achieve a high performance, high ON/OFF current ratio and low set voltage resistive switching device could hardly have been attained.

In this paper, we prepared Pt/Ag/ZnO:Li/Pt/Ti and Pt/Ag/ZnO/Pt/Ti resistive switching devices with a high resistive switching behavior by using magnetron sputtering method. Based on X-ray diffraction (XRD) and X-ray photoelectron spectroscopy (XPS) tests, the crystal structure and chemical composition of the Li-doped ZnO thin films were characterized, and the device resistive switching behaviors at a direct current (DC) voltage mode and a pulse mode were studied, respectively. Meanwhile, how the thickness of the thin films influences the characteristics of the device was investigated and the mechanism of the resistive switching behaviors for the proposed devices was also analyzed. This study provides a new strategy for fabricating high performance ReRAM device.

## 2. Experimental Details

### 2.1. Materials and Method

**Figure 1:**
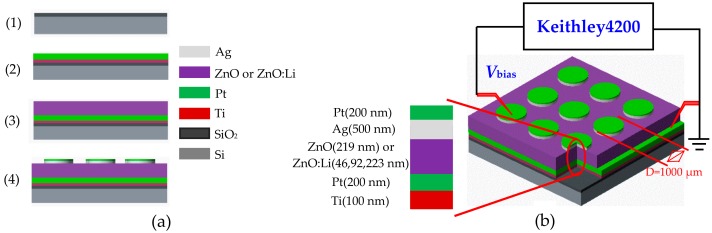
(**a**) Main fabrication process of Pt/Ag/ZnO:Li/Pt/Ti resistive switching device; (**b**) The basic structure and measurement configurations.

The Pt/Ag/ZnO:Li/Pt/Ti and Pt/Ag/ZnO/Pt/Ti resistive switching devices were fabricated by using radio frequency (RF) magnetron sputtering (JGP-DZS, Shenyang Sky Technology Development Co.Ltd, Shenyang, China) on SiO_2_/Si substrates. The main fabrication process of the proposed devices is shown in Figure 1a. (1) cleaning a silicon wafer substrate with <100> orientation by using standard cleaning method, growing oxide layer on the surface of the silicon wafer by thermal oxidation method to form a 200 nm thickness SiO_2_ layer; (2) depositing Ti thin films as adhesion layer on the SiO_2_/Si substrate based on a direct current RF magnetron sputtering method using a pure Ti target (size: D60 mm × 5 mm, purity: 99.99%), and then depositing Pt thin films above the resulted Ti thin films by repeating the above magnetron sputtering method (using a pure Pt target size: D60 mm × 5 mm, purity: 99.99%) to obtain the BE; (3) sputtering resistive switching layer by RF magnetron sputtering method using two ceramic targets, i.e., a pure ZnO (size: D60 mm × 4 mm, purity: 99.99%) and Li-doped ZnO (size: D60 mm × 4 mm, the Li content is 8 wt.% by Li_2_CO_3_ doped in the ZnO); (4) using a metal mask method to depositing Ag on the surface of ZnO thin films, and then a Pt layer with thickness of 200 nm was deposited on the Ag thin films in order to protect the TE from oxidation, where the sputtering Ti, Ag and Pt were carried out in atmosphere of argon (47 sccm) with RF power of 100 W and pressure of 1.0 Pa, but the pure ZnO and Li-doped ZnO was carried out in mixed atmosphere of argon (47 sccm) and oxygen (15 sccm) at 200 °C with RF power of 220 W and pressure of 1.0 Pa; Meanwhile, treating the Pt/Ag/ZnO:Li/Pt/Ti and Pt/Ag/ZnO/Pt/Ti resistive switching devices for 30 min by using alloying method to realize a complete Ohm contact.

Figure 1b shows the basic structure of the proposed resistive switching devices and the measurement configuration. The chip with a size of 2 × 2 cm^2^ is composed by 81 resistive switching units, where an inserted part cross-sectional view of each resistive unit is shown, consisting of three components including a Pt/Ti bottom electrode (BE), a resistive cell of pure ZnO or Li-doped ZnO thin films and a Pt/Ag top electrode (TE). A Ti adhesion layer with thickness of 100 nm and a Pt layer with thickness of 500 nm were deposited as the bottom electrode (BE), respectively. Thereafter, the ZnO or Li-doped ZnO thin films were sputtered on the BE. Finally, a 500 nm thickness Ag and a 200 nm thickness Pt top electrode (TE) with a diameter of 1000 μm were patterned using a mask technique. The resistive switching behaviors of the devices were measured using a semiconductor parameter analyzer (Keithley4200-SCS, Tektronix, Solon, OH, USA). In measurements of resistive switching, the BE was grounded, whereas the positive or negative voltage was applied on the TE by probe.

### 2.2. Analysis of ZnO Thin Films

**Figure 2:**
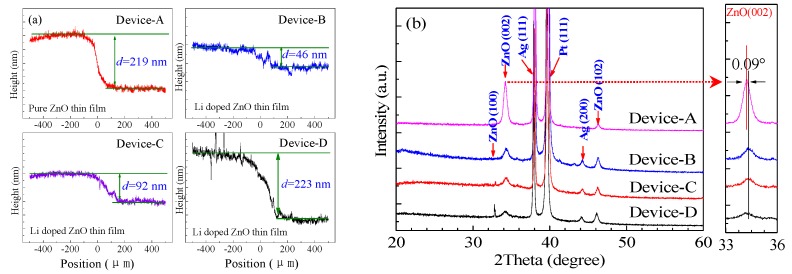
(**a**) The thicknesses of resistive switching layer; (**b**) XRD patterns of pure ZnO and Li-doped thin films, the inset shows magnified spectra near the ZnO (002) diffraction peak.

Based on the above fabrication process, two kinds of resistive switching devices were prepared. One is Pt/Ag/ZnO/Pt/Ti resistive switching device constructed by pure ZnO layer with thickness of 219 nm named as device-A, another is Pt/Ag/ZnO:Li/Pt/Ti devices named as device-B, device-C and device-D, composed Li-doped ZnO resistive switching layers with different thicknesses of 46 nm, 92 nm and 223 nm (measured by a step profiler NanoMap 500LS, AEP Technology, Santa Clara, CA, USA), respectively, as shown in Figure 2a.

To analyze the effects of Li-doped on the microstructures of the devices and observe the crystal structure change at different thin film thicknesses, the phase properties of the devices are tested by X-ray diffractometer (XRD, Bruker AXS D8 ADVANCE, Billerica, MA, USA) with Cu Kα1 radiation. Figure 2b presents the XRD patterns of the pure ZnO and Li-doped ZnO devices, representing the typical ZnO characteristic peaks of (100) and (102) planes (JCPDS No. 36-1451) as well as revealing the existence of a strong (002) orientation wurtzite structure. According to the pattern of the devices, the strong characteristic peaks representing Ag (111) and (200) appear at 38.02° and 44.2° [25], as well as the characteristic peak of Pt (111) at 39.03°, respectively. To further study the effect of Li-doped, an inset magnified pattern near the ZnO (002) peak is shown in Figure 2b, with a tiny peak right shift with 0.09° due to the small change of crystal lattice induced by doping Li into the ZnO thin films. Nevertheless, the crystal structure and growth direction of the Li-doped films are similar to the un-doped film [26]. Furthermore, the peaks of (100) and (102) at 32.85° and 44° are getting sharper with increasing the thicknesses of the Li-doped ZnO thin films, which may be attributed to the fact that Li can go to the interstitial and substitutional site in the lattice structure of ZnO, leading to the improved crystallinity of respective phases [27].

**Figure 3:**
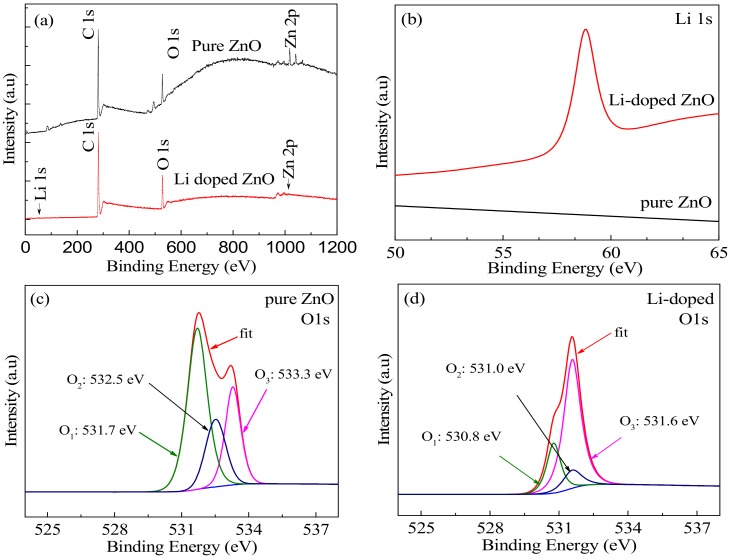
(**a**) XPS survey spectra of pure ZnO and Li-doped ZnO thin films; (**b**) XPS spectra of Li 1s; (**c**) XPS spectra of O 1s in pure ZnO thin film; (**d**) XPS spectra of O 1s in Li-doped ZnO thin films.

To further investigate the influence of Li doping on the chemical composition and valance state of the resulted ZnO thin films, the ZnO and Li-doped ZnO thin films with thicknesses of 219 nm and 223 nm were examined by X-ray photoelectron spectroscopy (XPS, VG ESCALAB MK II, VG Instruments, Manchester, UK), as shown in. A typical wide scan spectrum of ZnO films is shown in Figure 3, where all of the elements in the thin films are identified by a survey scan in the energy range from 0 to 1200 eV. The photoelectron peaks of the main elements, Zn 2p, O 1s, Li 1s and C 1s, where the C 1s peak comes from the adsorbed C on the surface of the sample. Based on the fitted XPS data of Li 1s in ZnO in Figure 3b and the quantitative analysis of the Li-doped ZnO thin film surface by XPS, Li concentration of 8.66 at % in ZnO thin films can be obtained.

Figure 3c,d show the fitted O 1s peak of pure ZnO and Li-doped ZnO thin films, respectively. The O 1s peak can be fitted into three Gaussian peaks with different binding energy components [28], including the lowest O_1_ peak, the medium O_2_ peak and the highest O_3_ peak, respectively representing the lattice oxygen in the wurtzite structure, the oxygen vacancies and chemisorbed oxygen on the surface of the ZnO thin films [29,30]. Figure 3c,d show the fitted O 1s peak of the two kinds of thin films under no surface pre-sputter, where O_iii_ can be ignored because there is a large amount of chemical absorption oxygen to appear on the surface of the thin films. Using Avantage software (ThermoFisher SCIENTIFIC, Waltham, MA, USA) to fit the O 1s, the percentages of the different types of oxygen in each film can be estimated by calculating the area of each peak [31]. Due to the reduced O_2_ region being relative to oxygen deficiency in the Li-doped ZnO thin films [32], it indicates the oxygen vacancies (V_O_) were decreased compared with that in the pure ZnO thin film. Thus, the defects in the ZnO thin films were reduced by using the Li doping, effectively improving the quality of the film and square resistance [33,34].

## 3. Results and Discussion

### 3.1. Resistive Switching Behavior under Direct Current Voltage

**Figure 4:**
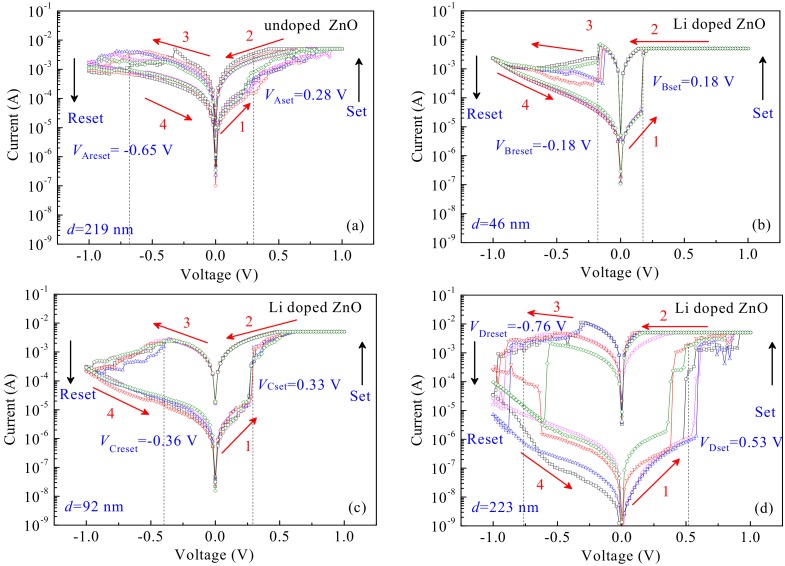
The *I*-*V* characteristics of devices at a DC voltage: (**a**) device-A; (**b**) device-B; (**c**) device-C; (**d**) device-D.

At room temperature, the resistive switching devices were measured by a semiconductor parameter analyzer under a direct current (DC) voltage sweep mode. Figure 4a–d show the current-voltage (*I*-*V*) characteristic curves of the devices-A, -B, -C and -D in 5 switching cycles, respectively (multiple cycles as seen Figure S1 in supporting information (SI)). A biased sweeping voltage was applied to the devices in a sequence of 0 V→1.0 V→0 V→−1.0 V→0 V. Initially, all of the devices stay at high resistance state (HRS) under no biased voltage. When exerting a positive biased voltage from 0 V to 1.0 V, a compliance current of 5.0 mA was restricted to prevent the devices from an irreversible breakdown. The device-A achieves an HRS to transform into a low resistance state (LRS) at an average threshold voltage of *V*_Aset_ = 0.28 V, with an ON/OFF current ratio (*I*_ON_/*I*_OFF_) of 10. In contrast, the device-B and -C with different thicknesses of thin films display clear bipolar resistive switching behaviors to transform an HRS into an LRS at lower average set voltages of *V*_Bset_ = 0.18 V and *V*_Cset_ = 0.33 V, both with *I*_ON_/*I*_OFF_ > 10^2^. With respect to the other devices, the device-D transforms from an HRS to an LRS at a higher average set voltage of *V*_Dset_ = 0.53 V and has a similar *I*_ON_/*I*_OFF_ to the device-B and -C, but achieving a bad resistance uniformity. When increasing the applied voltage up to a maximum of 1.0 V, and then gradually returning to 0 V, all of the devices still maintain in the LRS.

When applying a negative biased voltage from 0 V to −1.0 V (without a current compliance), the resistance of the devices transforms from an LRS to an HRS, taking as a reset operation process. From the Figure 4a–d, it can be seen that the device-A achieves a transformation from a LRS to a HRS at an high average reset voltage about *V*_Areset_ = −0.65 V, in contrast, the device-B and -C transform at lower average reset voltages of *V*_Breset_ = −0.18 V and *V*_Creset_ = −0.36 V, respectively. Nevertheless, the device-D transform at a higher average reset voltage *V*_Dreset_ = −0.76 V compared with device-A. When reversing the voltage from −1.0 V to 0 V, the HRS for the devices can be sustained. The test results show that all of the devices have good bipolar resistive switching characteristics, i.e., the device-A exhibits an analog resistive switching behavior with a small *I*_ON_/*I*_OFF_ ratio of ~10^0^, the device-B, -C and -D perform a digital resistive switching behavior with a large *I*_ON_/*I*_OFF_ ratio more than 10^2^. In addition, the device-C achieves good uniformity and stable LRS and HRS compared with the others, benefiting to the applications of resistive switching devices in non-volatile memories and logic operations. To investigate the uniformity of the resulted devices, the coefficient of variation (σ/μ) was calculated as shown in Figure S2a,b in SI. It can be seen that the device-C has relatively high slopes of *R*_on_ and *R*_off_ distributions and a small coefficient of variation σ/μ. In consequence, stable and uniform distributions of LRS and HRS comparing with the other devices can be obtained by the gradual SET and RESET operations, this can be attributed to the perpendicular ZnO grain orientation under Li-doped synergistic effect being beneficial for ensuring that the formation and rupture of the conducting filaments caused by the carriers drift along the (002) grain boundaries under an electric field [35]. Thus, it is possible for the device-C to realize a better high-switching uniformity of the LRS and HRS resistance, a high switching current ratio, low set and reset voltages.

**Figure 5:**
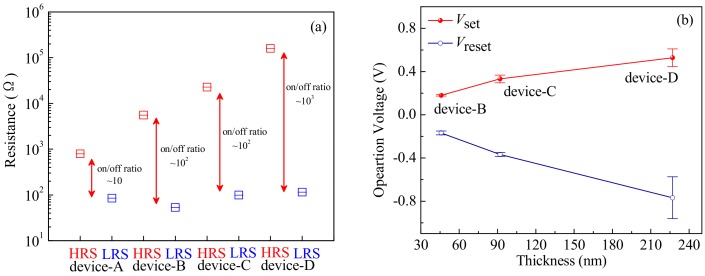
(**a**) Box plots of the high resistance state (HRS) and low resistance state (LRS) resistances of device-A, -B, -C and -D; (**b**) The switching voltage variation of device with different thickness Li-doped ZnO thin films.

To analyze the effect of Li-doped and thin film thicknesses on the resistive switching behaviors, the average values of *R*_HRS_ at an HRS, *R*_LRS_ at an LRS, *V*_set_ and *V*_reset_ for the fabricated devices are obtained by analyzing and calculating the data measured as shown in Figure 4. Figure 5a shows a comparison relationship of *R*_HRS_ and *R*_LRS_ of the devices at an HRS and an LRS. As can be seen from Figure 5a, the devices with Li-doped achieve a high ON/OFF current ratio *I*_ON_/*I*_OFF_ and a big *R*_HRS_ compared with that of the device-A. Moreover, with the increase of the thin film thicknesses, the *I*_ON_/*I*_OFF_ and *R*_HRS_ obviously increase, but slightly influence *R*_LRS_. In addition, the absolute values of the *V*_set_ and *V*_reset_ of devices-B, -C and -D also increase with the increase of the Li-doped ZnO thin film thicknesses, as shown in Figure 4b.

### 3.2. Resistive Switching Performance under Pulse Voltage

**Figure 6:**
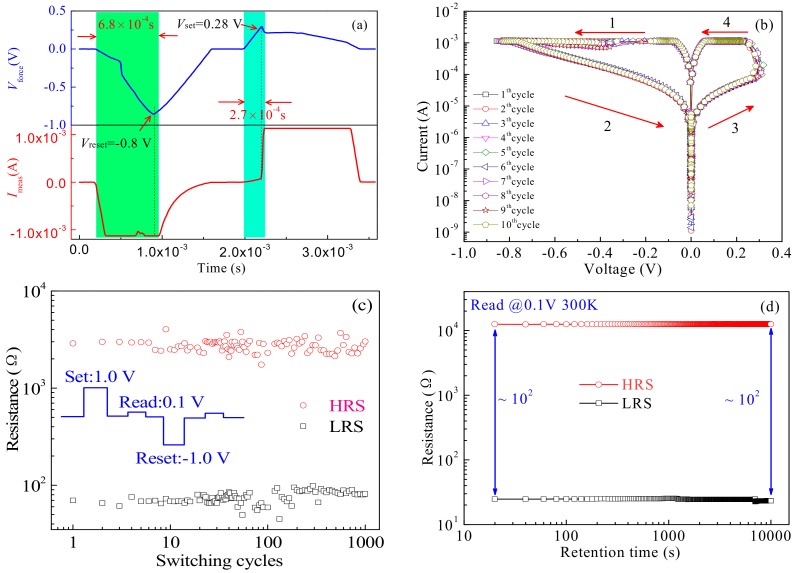
(**a**) The transient pulse characteristics of the device-C, the transition duration of set and reset processes is highlighted with greenish and bluish blocks, respectively; (**b**)The switching characteristics of devices-C at a pulse voltage, the pulse with a reset voltage of −1.0 V, a set pulse of 1.0 V, a width time of 1 × 10^−^^6^ s, rise time of 7 × 10^−4^ s and delay time of 2 × 10^−4^ s; (**c**) Endurance performance of devices-C; (**d**) Retention performance, indicating stable HRS and LRS up to 10^4^ s.

Based on the *I*-*V* characteristics of the devices under a DC voltage, it can be found that the device-C achieves a super resistive switching behavior comparing with the others. However, exploring the resistive switching behaviors at a pulse voltage is necessary to improve the properties of memristors such as low Joule heating effect, small current drift and excellent resistive performance [36]. Therefore, we further studied the resistive switching behavior of the device-C at a pulse voltage. The *V-t* and *I-t* characteristic tests of the device-C were carried out at 1.0 V and −1.0 V pulses, as shown in Figure 6a. The applied pulse voltage *V*_force_ (seen in the blue curve) and measured current *I*_meas_ (seen in the red curve) across the device-C are taken as a function relative to time, the reset and set details of the resistive switching behaviors are highlighted with green and blue regions in Figure 6a, respectively. When exerting the positive pulse voltage up to 0.28 V, the current of the device-C abruptly jumps, with an effective set pulse width less than 2.7 × 10^−4^ s. In response, the reset process occurs at a biased voltage of −0.8 V, with an effective set pulse width less than 6.8 × 10^−4^ s. Figure 6b shows the *I-V* characteristics of the device-C with a continuous 10 switching cycles at a pulse voltage. When exerting a reset pulse to the device-C, the current quickly decreases at a certain voltage of −0.8 V to transform from an LRS to an HRS. Correspondingly, the current of the device-C obviously jumps from an HRS to an LRS at a set of pulse voltage of 0.3 V. Based on the above similar test results in Figure 6a,b, it is possible for the device-C to transform from an HRS to an LRS at a pulse voltage.

The excellent endurance and the long retention belong to two fundamental properties in nonvolatile memory devices, i.e., performing a multiple erases/writes of data and remaining the data of memory device for a long time. Thus, the endurance measurements of the device-C were conducted by using a pulse and a nondisruptive memory measurement protocol as shown in Figure 6c. By applying a series of set pulses (1.0 V) and reset pulses (−1.0 V) to the devices, the resistance of the devices was measured at a read pulse of 0.1 V. It can be seen that the pulse widths both of the set and reset are 7 × 10^−4^ s, with a delay time of 1 × 10^−6^ s. Two distinct resolved resistance states with a remarkable resistive switching window (HRS/LRS) were observed for the device-C. It indicates that the proposed device-C can achieve an excellent endurance cycle of 10^3^ and a high ON/OFF current ratio *I*_ON_/*I*_OFF_ more than 10 at a read voltage of 0.1 V, which is necessary for practical memristor applications. Meanwhile, the stability at the LRS and HRS was investigated using a retention test at room temperature. Figure 6d shows the retention performance of the device-C, with a stability of LRS and HRS more than 10^4^ s without any observable degradation in the two states. The above measured results indicate that the device achieves a good endurance and a long retention property compared with that of the other devices (seen in Figure S3 in SI), being suitable to the practical applications of nonvolatile memory.

### 3.3. Mechanism Analysis of Resistive Switching

**Figure 7:**
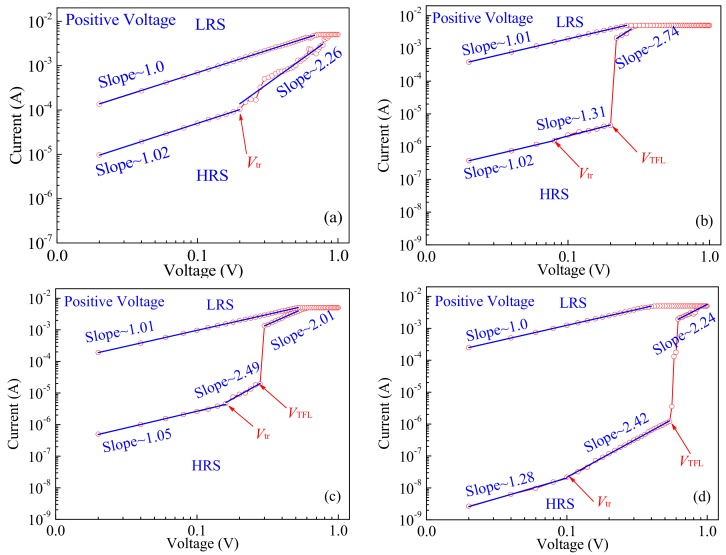
Logarithmic plot of the *I*-*V* curves for the HRS and LRS under the positive bias region: (**a**) The device-A; (**b**) The device-B; (**c**) The device-C; (**d**) The device-D.

To understand the conduction mechanisms of the resistive switching device, the *I-V* characteristic curves of all devices in the positive voltage regions are plotted in a double logarithmic scale as shown in Figure 7. The conduction mechanism of the resistive switching devices can be best understood by the space-charge-limited conduction (SCLC) model and conductive filament model [37,38]. The analog resistive switching mechanisms of device-A can be analyzed by Figure 7a. Under the low positive bias lower than the transition voltage *V*_tr_ at an HRS, the transport follows Ohm’s law (with slope of ~1)_._ When the applied voltage bias exceeds *V*_tr_, meaning that the conduction enters a trap-filled-limited region, the traps are gradual filled up and the conduction becomes space-charge-limited, following Child’s law (*I*_Child_ ∝*V*^2^). Based on the above analysis, due to pure ZnO thin films containing a large amount of oxygen vacancies, device-A represents an analog resistive switching behavior attributed to the charge trapping and de-trapping by oxygen vacancies [39]. With respect to that, the digital resistive switching mechanisms of device-B, C and D can be investigated by Figure 7b–d. Under an original low bias at an HRS, the transports of the Li-doped devices are in accordance with the above Ohm’s law with two different slopes. When the voltage bias reaches the trap-filled limit (TFL) voltage *V*_TFL_, all traps are filled up and the conduction becomes the SCLC model, implying that the density of thermally generated free carriers is larger than the injected carriers [40]. Meanwhile, Ag conductive filaments are constructed between the top and the bottom electrodes, leading to a current abrupt jump as a digital resistive switching behavior [41].

**Figure 8:**
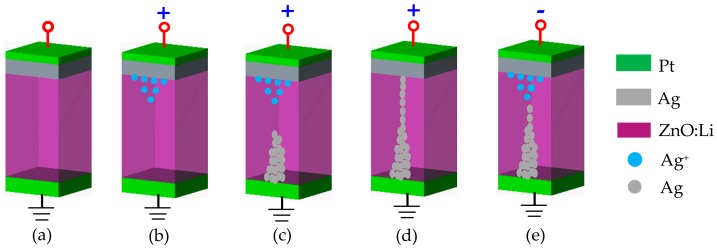
The schematic diagram for the mechanism of resistive switching effects in Pt/Ag/ZnO:Li/Pt/Ti devices: (**a**) Initial state; (**b**) The TE Ag loses electrons and migrates to the BE at a positive bias voltage; (**c**) Ag^+^ ions get electrons on the BE to become Ag atoms and grow toward the TE; (**d**) Forming conductive filaments; (**e**) When exerting a negative bias voltage, the conductive filament dissolves.

The above analysis indicates that the formation and rupture of the Ag based conduct filaments (CFs) are responsible for the resistive switching behaviors of devices [35]. The schematics of the resistive switching mechanism models under different applied DC voltages are shown in Figure 8. Figure 8a shows the initial state of the devices under no applied voltage. When exerting a positive voltage to the Ag TE, an oxidation reaction exists on this electrochemically active material, resulting in the generation and accumulation of Ag^+^ ions near the Ag electrode, as shown in Figure 8b. Initially, these Ag^+^ ions drift through the ZnO:Li thin films to BE (Pt), and then are reduced there due to the action of the external electric field, as shown in Figure 8c. The successive Ag atoms accumulate near the BE start to form a bridge connection with the TE in the ZnO:Li thin films. Finally, the conduct filament grows between the two electrodes, setting the system into an ON state corresponding to the LRS as shown in Figure 8d. When reversing the polarity of the applied voltage, an electrochemical dissolution takes place somewhere along the filament until almost completely dissolving [42], resetting the system into an OFF state corresponding to the HRS, as depicted in Figure 8e.

## 4. Conclusions

In summary, resistive switching devices Pt/Ag/ZnO:Li/Pt/Ti with different film thicknesses were fabricated by using magnetron sputtering method and mask technology. Comparing with the Pt/Ag/ZnO/Pt/Ti device, the proposed Pt/Ag/ZnO:Li/Pt/Ti devices achieve better bipolar resistive switching characteristics, including a high ON/OFF current ratio *I*_ON_/*I*_OFF_, a low set voltage (<1.0 V) and reset voltage (<−1.0 V). With the addition of the Li-doped ZnO thin film thicknesses, the ON/OFF current ratio *I*_ON_/*I*_OFF_ and the set voltage *V*_set_ of the devices increase. In addition, the resistive switching devices can quickly transform from an HRS to an LRS, with an excellent endurance more than 10^3^ cycles at an applied pulse voltage and a long retention up to 10^4^ s. By analyzing the *I*-*V* characteristics and installing conduction models, the relative resistive switching mechanism of the devices was investigated based on analog and digital switching characteristics. This study on the ZnO:Li thin film device makes it possible to expand the future practical memory device applications. 

## Supplementary Materials

The following are available online at https://www.mdpi.com/1996-1944/12/8/1282/s1, Figure S1: The I-V characteristics: (a) device-A; (b) device-B; (c) device-C; (d) device-D, Figure S2: (a) The cumulative distributions of *R*_LRS_ and *R*_HRS_; (b) The coefficient of variation of *R*_LRS_ and *R*_HRS_ distribution, Figure S3: The endurance performances of (a) device-A; (b) device-B; (c) device-D.
