# Supplementary material for: Resistive Switching Characteristics of Li-Doped ZnO Thin Films Based on Magnetron Sputtering
Source: Materials (Basel). 2019 Apr 18;12(8):1282. doi: 10.3390/ma12081282 (PMC6515171; doi:10.3390/ma12081282)
Supplement: Supplementary file 1 [file materials-12-01282-s001.pdf]

Supporting Information

"Resistive Switching Characteristics of Li-doped ZnO Thin Films based on Magnetron Sputtering"

1. Multiple *I-V* characteristics of the resulted devices under a DC voltage.

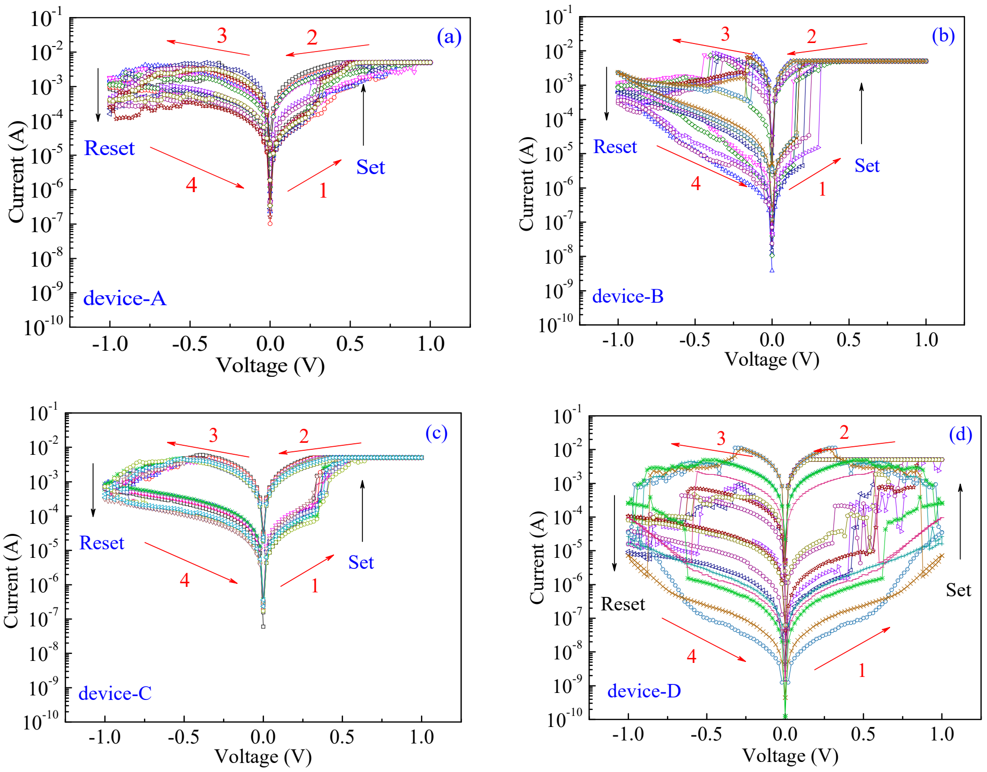

Figure S1. The *I-V* characteristics: (a) device-A; (b) device-B; (c) device-C; (d) device-D.

2. The curves of cumulative distributions and the coefficient of variation (σ/μ) of R_{LRS} and R_{HRS}

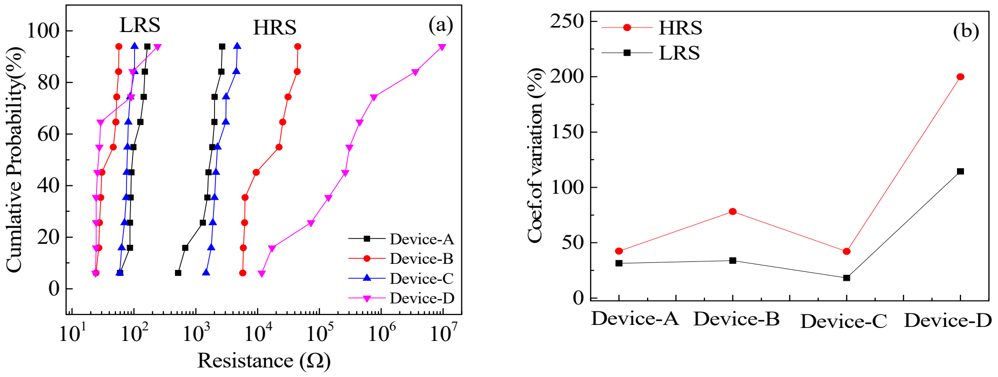

Figure S2. (a) The cumulative distributions of R_{LRS} and R_{HRS}; (b) The coefficient of

variation of R_{LRS} and R_{HRS} distribution.

3. The endurance characteristics of resistive switching device.

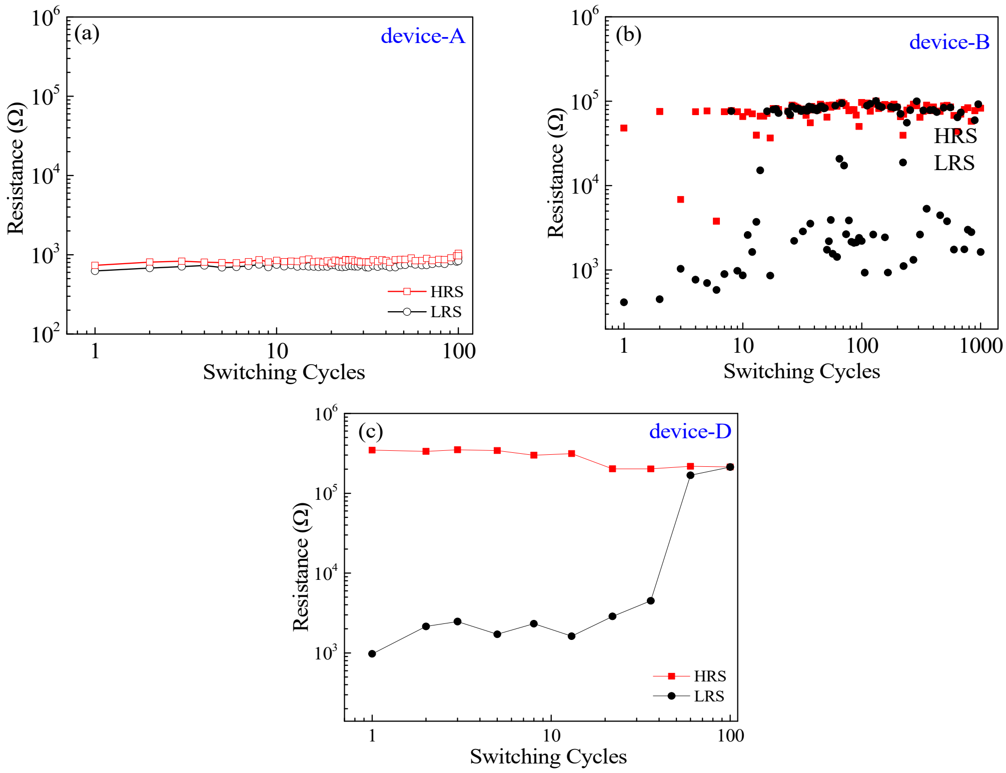

Figure S3. The endurance performances of (a) device-A; (b) device-B; (c) device-D.
